# Quantitative real-time RT-PCR and chromogenic *in situ *hybridization: precise methods to detect HER-2 status in breast carcinoma

**DOI:** 10.1186/1471-2407-9-90

**Published:** 2009-03-23

**Authors:** Fabíola E Rosa, Sara M Silveira, Cássia GT Silveira, Nádia A Bérgamo, Francisco A Moraes Neto, Maria AC Domingues, Fernando A Soares, José RF Caldeira, Silvia R Rogatto

**Affiliations:** 1 Department of Genetics, Institute of Biosciences, UNESP – São Paulo State University, Botucatu, Sao Paulo, Brazil; 2 Department of Urology, Faculty of Medicine, UNESP – São Paulo State University, Botucatu, Brazil; 3 Department of Pathology, Amaral Carvalho Hospital, Jaú, São Paulo, Brazil; 4 Department of Pathology, Faculty of Medicine, UNESP – São Paulo State University, Botucatu, São Paulo, Brazil; 5 Department of Pathology, Fundação Antonio Prudente, Hospital AC Camargo, São Paulo, Brazil; 6 Department of Senology, Amaral Carvalho Hospital, Jaú, São Paulo, Brazil; 7 NeoGene Laboratory, Fundação Antonio Prudente 211, Hospital AC Camargo – Liberdade São Paulo, Brazil

## Abstract

**Background:**

*HER-2 *gene testing has become an integral part of breast cancer patient diagnosis. The most commonly used assay in the clinical setting for evaluating HER-2 status is immunohistochemistry (IHC) and fluorescence *in situ *hybridization (FISH). These procedures permit correlation between *HER-2 *expression and morphological features. However, FISH signals are labile and fade over time, making post-revision of the tumor difficult. CISH (chromogenic *in situ *hybridization) is an alternative procedure, with certain advantages, although still limited as a diagnostic tool in breast carcinomas.

**Methods:**

To elucidate the molecular profile of HER-2 status, mRNA and protein expression in 75 invasive breast carcinomas were analyzed by real time quantitative RT-PCR (qRT-PCR) and IHC, respectively. Amplifications were evaluated in 43 of these cases by CISH and in 11 by FISH.

**Results:**

The concordance rate between IHC and qRT-PCR results was 78.9%, and 94.6% for qRT-PCR and CISH. Intratumoral heterogeneity of *HER-2 *status was identified in three cases by CISH. The results of the three procedures were compared and showed a concordance rate of 83.8%; higher discordances were observed in 0 or 1+ immunostaining cases, which showed high-level amplification (15.4%) and *HER-2 *transcript overexpression (20%). Moreover, 2+ immunostaining cases presented nonamplified status (50%) by CISH and *HER-2 *downexpression (38.5%) by qRT-PCR. In general, concordance occurred between qRT-PCR and CISH results. A high concordance was observed between CISH/qRT-PCR and FISH. Comparisons with clinicopathological data revealed a significant association between *HER-2 *downexpression and the involvement of less than four lymph nodes (*P *= 0.0350).

**Conclusion:**

Based on these findings, qRT-PCR was more precise and reproducible than IHC. Furthermore, CISH was revealed as an alternative and useful procedure for investigating amplifications involving the *HER-2 *gene.

## Background

In breast cancer, the assays routinely used in clinical practice are those that address a specific management decision. Hormonal therapy is based on estrogen receptor (ESR) and progesterone receptor (PGR) status. Trastuzumab (Herceptin™, Genentech, Inc, San Francisco, CA, USA) therapy, a humanized monoclonal antibody, is based on HER-2 status.

*ERBB2/HER-2 *(*HER-2/neu*, *NEU*, *NGL*, *HER2*, *TKR1*, *CD340*) encodes a membrane receptor protein in the growth factor receptor gene family presenting tyrosine kinase activity [1,2]. *HER-2 *plays a role in the pathogenesis of a significant number of human tumors. It is altered in approximately 20–30% of breast carcinomas and this is manifested as gene amplification and/or protein overexpression [3-5]. These alterations are associated with a shorter disease free period and overall survival and with resistance to tamoxifen antiestrogen therapy and other chemotherapy regimens, regardless of the nodal or hormone receptor status [3,4,6,7]. Moreover, breast carcinoma patients presenting *HER-2 *amplification or overexpression can benefit from anthracycline-based regimens, as well as trastuzumab [8].

The therapy choice for breast cancer patients depends on the discrimination of HER-2 status. Reliable laboratory data in evaluating HER-2 status is essential, because the treatment is beneficial for advanced breast cancer and avoids potential cardiotoxic effects in women not showing amplification and overexpression [9]. The most commonly used assay in the clinical setting for evaluating HER-2 status is immunohistochemistry (IHC) and fluorescence *in situ *hybridization (FISH), both approved by the FDA (U.S. Food and Drug Administration). More recently, the CISH (chromogenic *in situ *hybridization) methodology, also approved by FDA, has emerged as a potential alternative to FISH. When compared with FISH, CISH has been described as having several advantages. CISH does not require an expensive fluorescence microscope with multi-band-pass filters, it produces a permanent staining and samples can be archived indefinitely, thus avoiding archival recording with an expensive CDD camera. The morphology is easier to analyze, particularly for distinguishing invasive cancer cells and *in situ *components. Moreover, tumor heterogeneity is promptly identified, even at low magnification (20×) [10-13]. In FISH analysis, tissue morphology and gene amplification are primarily disconnected because of tumor cells for signal evaluation are based on nuclear DAPI (4', 6-Diamidino-2-phenylindole) or propidium iodide staining, which does not always permit adequate histopathological evaluation of the cells [14]. CISH is a useful methodology for confirming ambiguous IHC results [11]. In addition, polymerase chain reaction (PCR) based technology has been demonstrated to successfully evaluate specific mRNAs, especially those present in low copy numbers in a small number of cells or in small quantities of tissue, and mRNAs expressed in mixed-cell populations. Quantitative real time reverse transcript PCR (qRT-PCR) is a quantitative method easily amenable to standardization. However, qRT-PCR suffers from the same drawback as other PCR-based methods. The tumor cell population within the tissue under evaluation must be isolated, the template quality, operator variability, subjectivity in data analysis and reporting are technical aspects that must be considered [14,15]. This procedure is an alternative for scoring *HER-2 *status in human breast cancer. Limitations based on tumor heterogeneity and amplification of *HER-2 *in noninvasive cancer can be eliminated by the use of laser microdissection, although this seems to be impracticable for routine diagnosis [14].

The purpose of the current study was to assess agreement between gene amplification detection by CISH and transcript (qRT-PCR) and protein (IHC) expression, as well as to evaluate their relevance for determining *HER-2 *status in breast carcinomas. In addition, the data were correlated with clinicopathological features, such as tumor size, lymph node status, histological grade and Ki-67 status.

## Methods

### Patients

Seventy-five breast carcinoma samples were obtained between 2000 and 2004, from Amaral Carvalho Hospital, Jaú (SP, Brazil). The patients were accrued consecutively and the criterion for inclusion in the study was no previous histological diagnosis of breast cancer. Patients underwent segmental resection or mastectomy and none of them had received radiotherapy or chemotherapy prior to surgery. All patients were advised of the procedures and provided written informed consent. The Ethics Committee from Amaral Carvalho Hospital Foundation approved this study (CEPFHAC 007/05).

Seventy tumors were infiltrating ductal and five infiltrating lobular carcinomas, in which most presented operable stage II and III breast cancer and positive axillary lymph nodes. Most patients were more than 50 years-old (64%) with a mean of 58 ± 15.5 years (range, 30–94 years); most tumors were more than 2 cm (73.3%) and half of them showed low Ki-67 positivity. The mean follow-up was 49.8 ± 20.1 months (varying between 23 to 83 months). During this interval, five patients died due to unrelated causes, two patients presented recurrence in the same breast, eight presented metastasis (two spreading to the bone and six to the lung) and six missed their follow-ups. Patients with a family history of cancer were noted, particularly among first-degree and second-degree relatives, and whenever possible, the cancer was confirmed with documented medical records or ascertained from the death certificate (Table 1). The patients received different chemotherapy treatments: AC (adriamycin, cyclophosphamide); FEC (5-fluorouracil, 4-epirubicin, cyclophosphamide); FAC (5-fluorouracil, adriamycin, cyclophosphamide); CMF (cyclophosphamide, methotrexate, 5-fluorouracil), at 100% or 70% of the dose, according to the age and clinical status of the patient. Fifty-five patients (73%) received radiotherapy and 51 ESR1 positive and/or PGR positive cases (68%), as determined by IHC analysis, were treated with tamoxifen (20 mg/day) for 60 months at the end of the chemotherapy treatment. Some patients presenting intolerance or side effects (postmenopausal) were treated with aromatase inhibitors, such as anastrozole (1 mg/day) or letrozole (2.5 mg/day).

**Table 1:** Comparison of clinicopathological features with *HER-2 *status using CISH, qRT-PCR (qPCR), and IHC.

Variables (n)^a^	CISH		P *	qPCR		P *	IHC		P *
				
	≤ **5**	>10		≤ 2.00	>2.00		0/1+	2/3+	
**Age (years)**									
≤ 50 (28)	9	5	0.9541	15	13	0.2569	17	11	0.3651
>50 (48)	15	8		32	16		34	14	
									
**Lymph node status**									
<4 (57)	21	9	0.1756	38	19	0.0915	40	17	0.4723
≥ 4 (32)	3	4		8	10		11	7	
ND (1)	0	0		0	1		0	1	
									
**Tumor Size (cm)**									
0 – 2 (19)	5	5	0.1883	10	9	0.2953	11	8	0.2745
>2 (56)	19	7		37	19		40	16	
ND (1)	0	1		0	1		0	1	
									
**Clinical Stage**									
I/IIA/IIB (65)	21	9	0.1756	41	24	0.5901	44	21	0.7912
IIIA/IIIB (11)	3	4		6	5		7	4	
									
**Histologic grade**									
I/II (36)	10	6	1.000	22	14	0.9237	23	13	0.6767
III (35)	10	6		21	14		24	11	
ND (5)	4	1		4	1		4	1	
									
**Ki-67 status**									
Low (≤ 25%) (32)	11	6	0.5133	19	13	0.5354	21	11	0.7209
High (> 25%) (31)	8	7		16	15		19	12	
Not reactive (13)	5	0		12	1		11	2	
									
**Familial History of Cancer**									
Yes (15)	4	3	0.6346	7	8	0.1769	10	5	0.9678
No (61)	20	10		40	21		41	20	

^a ^Number of the cases evaluated by qRT-PCR and IHC, while 37 cases were evaluated using CISH.

* Chi-Square test.

ND: not determined.

Histopathological classification was performed according to the WHO International Classification of Disease for Oncology [16] and clinical stage was determined according to the UICC TNM classification [17]. The malignancy of infiltrating carcinomas was scored according to the Scarff-Bloom and Richardson grading system [18].

### HER-2 copy number alterations

In 43 out of 75 samples that presented available histological sections, CISH analysis was performed (32 additional cases are part of the Hospital's sample bank especially devoted to diagnosis). Eleven cases that presented adequate tumor samples on slides were evaluated by dual color FISH. CISH and FISH were carried out on 4 μm thick archival formalin fixed paraffin embedded tumour samples using a Zymed SPoT-Light HER2 CISH Kit (Zymed Laboratories Inc, San Francisco, CA) and a *HER2 *FISH pharmDx^TM ^Kit (DakoCytomation, Denmark), respectively, according to manufacturers' instructions. At least 200 non-overlapping tumor cell nuclei were evaluated by CISH. According to the manufacturer's instructions, the tumors were classified depending on the number of *HER-2 *gene copies in the nuclei as: (a) nonamplified, those tumor cells with two to five brown intranuclear spots per nucleus; (b) low-level amplification, when six to 10 signals per nucleus were detected in more than 50% of tumor cells or when a small coalescing signal cluster was found; (c) high-level amplification, defined as more than 10 copies per nucleus or when copy clusters were observed in more than 50% of cancer cells.

By FISH, *HER-2 *and chromosome 17 centromere signals were counted in at least 60 nuclei; a *HER-2*/CEP-17 ratio ≥ 2.0 was considered positive for *HER-2 *gene amplification [19]. A fluorescence microscope (Olympus AX61, Olympus Optical, Hamburg, Germany), equipped with a CCD camera (Photometrics CH 250, Huntington Beach, CA) was used. Image analysis was performed with the software Applied Spectral Imaging CGH View 3.0 (Olympus).

Slides from both procedures were randomly distributed to three independent blinded observers (SMS, CGTS, and NAB). Any discrepancy in sample classification was addressed by immediate review and the final result was reached by consensus. In addition, different individually identified tumor areas were analyzed.

### Isolation of tumoral cells by microdissection

Immediately after surgery, the tumor samples were frozen at -80°C. Eight successive unstained slides from frozen samples were prepared using a cryostat and stored at -80°C. The first and the last slides were stained with hematoxylin-eosin for histopathological evaluation. Tumoral components were precisely outlined and labeled under the microscope. The defined areas were retrieved by manual microdissection. To minimize dilution of the PCR signal by nontumoral and nonamplified cells, sections containing at least 90% tumor cells were selected for RNA extraction.

### Total RNA isolation and reverse transcription

Total RNA was extracted from pulverized frozen tumor tissue using the Rneasy mini Kit (Qiagen GmbH, Hilden, Germany), according to the manufacturer's instructions. Reverse transcription using SuperScript™ II reverse transcriptase (Invitrogen Life Technologies Inc., Carlsbad, CA) was carried out for 60 min at 42°C and the reaction mixture was subsequently inactivated for 15 min at 70°C as previously described [20]. The cDNA was stored at -70°C.

### Real time quantitative RT-PCR (qRT-PCR)

Seventy-five breast carcinomas were evaluated by qRT-PCR. Four normal breast tissue samples from patients who underwent mammary reduction and confirmed as histopathologically normal were used as controls. PCR amplification was performed in an ABI Prism 7000 Sequence Detection System (Applied Biosystems, Foster City, CA, USA). Primers and TaqMan probes for *HER-2 *and the *GAPDH *control reference gene were designed and synthesized according to Taqman Gene Expression Assay (assays Hs00170433_m1 and 4326317E, respectively) (Applied Biosystems, Foster City, CA, USA). Quantitative data was analyzed using the Sequence Detection System software (v1.0; Applied Biosystems). PCR reactions were carried out in a total volume of 10 μL, according the manufacturer's instructions. A relative standard curve was constructed for all primers with serial dilutions of placenta cDNA (100, 50, 25, 12.5, and 8 ng/uL). The standard curves of the target and reference genes showed similar results of efficacy (>90%). The reactions were performed in duplicate. The relative quantification (RQ) was given by the ratio between the mean value of the target gene and the mean value of the reference gene (*GAPDH*) in each sample. The relative amount of PCR product generated from each primer set was determined on the basis of the cycle threshold (Ct) value. The RQ was calculated by 2^-ΔΔCT ^[21]. *HER-2 *relative expression level was compared with the ratio of healthy controls. Overexpression was defined as the mean *HER-2*/reference gene ratio RQ>2.00.

### Immunohistochemistry (IHC)

HER-2 protein levels were performed using rabbit monoclonal antibody (SP3 Clone) (Thermo Fisher Scientific, Fremont, CA, USA) (dilution 1:100) in 75 cases. After incubation for one hour, the sections were washed in PBS, incubated for 30 min with secondary biotinylated antibody and treated for 30 min with streptavidin peroxidase complex (LSAB, DAKO, Carpinteria, CA, USA). The sections were developed with 3,3'-diaminobenzidine (DAB) and counterstained with hematoxylin. Negative and positive control slides were included in each assay. The results were scored as: (0) no immunoreactivity; (1+) weak and incomplete immunoreactivity; (2+) weak and complete membrane immunoreactivity in more than 10% of the tumor cells or strong and complete membrane immunoreactivity in less than 10% of the tumor cells; and (3+) strong and complete membrane immunoreactivity in more than 10% of the tumor cells. Slides were randomly distributed to two independent blinded observers (FAMN and MACD). Only the invasive component of the neoplasia was assessed and scored. The level of Ki-67 was evaluated in all cases, but 13 cases were nonreactive. Ki-67, ESR1, and PGR protein expression was performed as described by Rosa et al. [20].

### Statistical Analysis

Comparisons between *HER-2 *expression (qRT-PCR and IHC), gene copy number (CISH) and several clinicopathological features were calculated using the Chi square test. Six patients that missed follow-up were censored as survivors in the statistical calculations. The correlation between the three methodologies was evaluated using One-way Analysis of Variance (ANOVA); differences were tested for significance by the Mann-Whitney test for two categories and by the Kruskal-Wallis test for three categories. Statistical significance was designated at *P *< 0.05. The concordance rate was obtained considering: (a) amplification by CISH *and *transcript overexpression (RQ>2.00) *and *2+ or 3+ immunostaining; (b) nonamplification by CISH *and *transcript downexpression (RQ ≤ 2.00) *and *0 or 1+ immunostaining.

## Results

**Figure 1:**
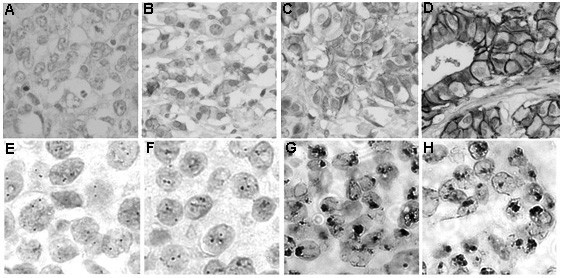
**Breast cancer cells showing immunohistochemistry and chromogen *in situ *hybridization results of *HER-2***. (A-D) IHC: HER-2 protein expression scored as 0 (A), 1+ (B), 2+ (C), and 3+ (D); (E-H) CISH: *HER-2 *gene detected in nuclei with two signals (E), more than two signals (F), and high-level amplification (G-H).

Breast cancer samples were assessed by *HER-2 *gene amplification and protein expression in histological samples using CISH and IHC, respectively (Figure 1). Transcript expression was evaluated by qRT-PCR in fresh samples after microdissection. *HER-2 *data obtained in all the procedures are shown in Tables 2 and 3.

**Table 2:** Comparison between protein expression by IHC and gene amplification by CISH and FISH.

	CISH (n = 37)		FISH (n = 8)	
	
IHC	No amplification (%)	High amplification (%)	No amplification (%)	Amplification (%)
0 or 1+	22 (84.6)	4 (15.4)	0 (0.0)	0 (0.0)
2+	2 (50.0)	2 (50.0)	0 (0.0)	2 (25.0)
3+	0 (0.0)	7 (100)	0 (0.0)	6 (75.0)

Total	24	13	0	8

**Table 3:** Transcript expression by qRT-PCR in relation to protein expression and gene amplification using IHC and CISH/FISH methodologies, respectively.

		qRT-PCR			
		
	n (%)	*HER-2 *ratio (range) *	*P*	Ratio ≤ 2.00 n (%)	Ratio >2.00 n (%)
**IHC**					
0 or 1+	50 (66.7)	0.93 (0.05–12.24)	<0.0001^a^	40 (80.0)	10 (20.0)
2+	12 (17.3)	2.46 (1.15–7.61)		5 (41.7)	7 (58.3)
3+	13 (16.0)	7.55 (1.07–20.43)		1 (7.7)	12 (92.3)
Total	75				
					
**CISH**					
No amplification	24 (64.9)	0.75 (0.05–1.95)	<0.0001^b^	24 (100)	0 (0.0)
High amplification	13 (35.1)	4.69 (1.07–20.43)		2 (15.4)	11 (84.6)
Total	37				
					
**FISH**					
No amplification	0 (0.0)	0 (0.0)	ND	0 (0.0)	0 (0.0)
Amplification	8 (100)	7.93 (2.64–20.43)		0 (0.0)	8 (100)
Total	8				

* Mean *HER-2*/reference gene ratios; ND: not determined.

^a ^Kruskal-Wallis test.

^b ^Mann-Whitney test.

**Figure 2:**
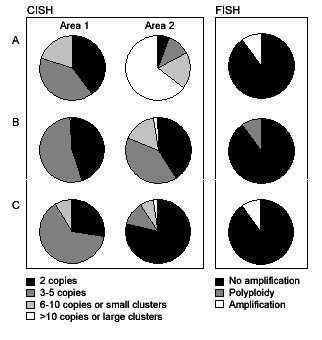
**Intratumoral heterogeneity of HER-2 gene status detected by chromogen *in situ *hybridization in two different areas (areas 1 and 2) from three breast tumors (A, B, and C)**. Nonamplified *HER-2 *gene (2–5 copies per nucleus), low-level amplification (6–10 copies or small clusters) and high-level amplification (>10 copies or large clusters) were observed in different areas from the same tumor. FISH results of the same cases are represented on the right side of the figure.

CISH was performed on 43 cytological samples; in three of these cases the spots presented inconclusive results; the brown spots were undistinguished. Twenty-four out of 37 tumors (64.9%) were not amplified by CISH, while 13 samples (35.1%) showed high-level *HER-2 *amplification. Different areas of the tumor were evaluated and the final analysis showed agreement, except in three cases (Figure 2A–C). Case A presented an equivalent number of nonamplified cells (2 copies and 3–5 copies) in area 1 and more than 50% of the cells with high-level amplification in area 2 (this case presented 0 score by IHC and normal expression level by qRT-PCR). Case B showed nonamplified cells in both areas, but in area 2, low-level and high-level amplification cells were also observed (IHC, 0 score; qRT-PCR, overexpression). Case C showed preferentially nonamplified cells; however, in area 1, cells with 3–5 copies were predominant and in area 2, a prevalence of two *HER-2 *copies was detected and the presence of sporadic cells with high-level amplification was also found (IHC, 3+ score; qRT-PCR, overexpression). More than 400 cells were evaluated for each of these cases. In the other samples (37 cases), the nuclear features were maintained, the morphological details were apparent and large gene copy clusters were easily detected at low magnification. Normal epithelial cells and lymphocytes showed one or two *HER-2 *signals per nucleus.

*HER-2 *amplification status by FISH was evaluated in 11 out of 43 cases investigated by CISH. The comparison between CISH and FISH revealed agreement in eight cases that presented high-level *HER-2 *amplification. These cases presented scores 2+ (two cases) and 3+ (six cases) by IHC. Only one of them, identified as amplified by CISH, showed sporadic cells (<10% of the cells) with polyploidy. The three cases that presented heterogeneity in two different areas of the tumor by CISH were also evaluated by FISH (Figure 2A–C). Case A presented similar results to that observed in the area 1 detected by CISH; amplification was observed in only 10% of the cells. Cases B and C showed principally no amplification by FISH, comparable to that detected by CISH (case B: 90% of the tumoral cells were not amplified and 10% presented polyploidy; case C: no amplification in 90% of the cells and amplification in 10%). An overall concordance between CISH and FISH results was found.

*HER-2 *relative expression level was evaluated in 75 samples by qRT-PCR in comparison with four healthy breast tissue and ranged between RQ = 0.05 to 20.43. Overexpression (RQ>2.00) was observed in 29 out of 75 cases (38.7%).

Among the 75 cases analyzed by IHC, 50 (66.7%) presented a score of 0 or 1+. The 2+ immunostaining cases comprised 13 samples (17.3%) and 12 cases (16.0%) presented 3+ scoring.

### CISH/FISH compared to IHC analysis

Four out of 26 cases (15.4%) presenting 0 or 1+ immunostaining scores showed high-level amplification by CISH. Two out of four samples (50%) scored as 2+ presented high-level amplification by CISH. All cases presenting 3+ scoring showed amplification by CISH (Table 2). The concordance rate between CISH and IHC was 83.8% (31 cases). Eight cases scored as 2+/3+ evaluated by FISH showed amplification.

### qRT-PCR compared to IHC results

**Figure 3:**
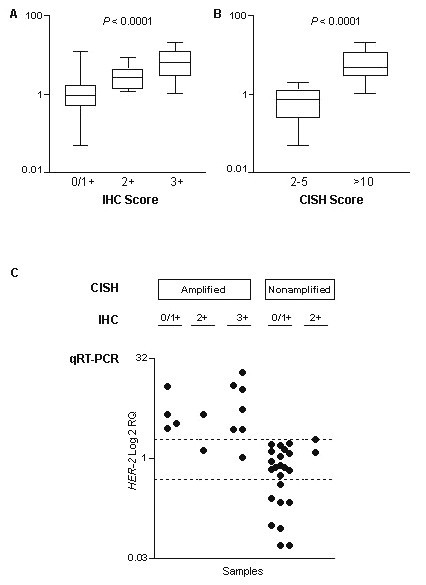
**(A) Association between transcript and protein (1+, 2+, and 3+) expression levels; (B) Correlation between transcript expression level and amplification (2–5 copies and >10 copies or large clusters); (C) CISH, IHC and qRT-PCR results in 37 samples of breast cancer**. The samples are indicated in dark circles. The transcript expression values by qRT-PCR are indicated in a log scale. Bars indicate the median value. *P *values are shown.

*HER-2 *transcript levels were significantly lower in cases presenting low protein expression (0 or 1+) than in cases presenting high expression (3+) (Figure 3A). Ten out of 50 cases (20%) presenting a score of 0 or 1+ showed overexpression by qRT-PCR. Seven out of 12 samples (58.3%) comprising 2+ immunostaining showed overexpression by qRT-PCR. Twelve out of 13 cases (92.3%) which presented a score of 3+ overexpressed *HER-2 *transcript (Table 3). Concordance between qRT-PCR and IHC was observed in 59 cases (78.7%).

### qRT-PCR compared to CISH/FISH analysis

Concordance between qRT-PCR and CISH was observed in 35 cases (94.6%). *HER-2 *mRNA levels were significantly lower in nonamplified cases by CISH (Figure 3B). None of the 24 nonamplified cases by CISH showed *HER-2 *overexpression. Eleven out of 13 cases (84.6%) presented both, high-level amplification and *HER-2 *overexpression by qRT-PCR. All cases showed *HER-2 *overexpression and amplification by FISH (Table 3).

Among the 37 cases evaluated by all three methodologies (CISH, qRT-PCR, and IHC), 31 (83.8%) showed concordance. Discrepancies were found in six cases; four cases 0 or 1+ by IHC showed *HER-2 *gene overexpression and were amplified by CISH; and two cases 2+ by IHC showed *HER-2 *gene RQ ≤ 2.00 and were not amplified by CISH (Figure 3C).

### Clinico-pathological data in comparison with HER-2 status

**Figure 4:**
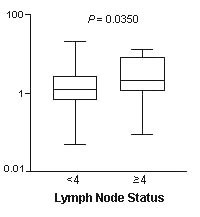
**Comparison between *HER-2 *transcript expression and lymph node involvement (<4 nodes and ≥ 4 nodes)**. Bars indicate the median value. *P *value is shown.

*HER-2 *data were also compared to clinicopathological features (Table 1). No statistical correlation was observed with age, tumor size, clinical stage, histological grade, Ki-67 status or familial history of cancer. A marginally significant correlation with lymph node status was detected: 82.6% of the cases presenting RQ ≤ 2.00 showed less than four positive lymph nodes (*P *= 0.0915). Comparison of individual *HER-2 *relative quantification values between the two classes of lymph node status (<4 and ≥ 4) showed a significant correlation (*P *= 0.0350), confirming the association between cases presenting RQ ≤ 2.00 and the involvement of less than four positive lymph nodes (Figure 4).

In the four lobular carcinomas evaluated by all three methodologies (CISH, qRT-PCR, and IHC), two presented discordant results: one case showed high-level amplification by CISH, *HER-2 *overexpression by qRT-PCR and negative immunostaining; the other sample presented nonamplified by CISH, RQ ≤ 2.00 by qRT-PCR and 2+ immunostaining by IHC analysis. The two remaining cases were nonamplified, RQ ≤ 2.00 and negative immunostaining. The case evaluated exclusively by qRT-PCR and IHC presented RQ ≤ 2.00 and negative immunostaining. Individual values of *HER-2 *expression by qRT-PCR were compared between ductal and lobular carcinomas and no significant association was observed (median RQ = 1.34 ± 3.83 and RQ = 1.7 ± 1.02, respectively; *P *= 0.7375, data not shown).

## Discussion

*HER-2 *gene has been extensively studied as a prognostic and predictive marker in clinical breast cancer, making this receptor a valuable target for the treatment of human breast cancer [22]. HER-2 status is predominantly evaluated by IHC staining, because it is easy to perform and presents a relatively low cost. However, a wide range of sensitivity and specificity was observed among various commercially available antibodies [23]. In addition, FISH is used for those cases that are scored as 2+. The advantage of FISH testing is that the quantitative interpretation of results with experience is relatively straightforward and concordance rates among observers are higher than with IHC in some studies [for review, [19]]. CISH is an alternative method to evaluate amplifications that requires a conventional light microscopy, permits a more rapid interpretation time and a review of the morphological details. A further advantage of CISH is that the probe signals are permanent and the slides can therefore be archived for long periods of time [24]. Data do not clearly demonstrate the superiority of either IHC or *in situ *hybridization (ISH) as a predictor of beneficial effects from anti-*HER-2 *therapy after validated testing has been carefully performed [25]. Quantitative real time PCR has the potential to become standard in terms of its performance, accuracy, sensitivity, broad dynamic range and high throughput capacity [26,27].

Concordance between FISH and CISH was 100% for the eight cases analyzed. This high concordance was also found by several other studies [11,14,28]. One of the eight cases displayed a small frequency of cells showing chromosome 17 polysomy by FISH and showed a score 2+ by IHC. Peiró et al. [29] showed that all of the polyploidy tumors analyzed presented 2+ immunostaining. Although a limited number of cases were evaluated by FISH in the present study, the data are in agreement with other studies which indicate that the chromogenic ISH technique seems to be sensitive and specific for the detection of *HER-2 *amplification in human archival tumor samples [14,28].

CISH analysis revealed intratumoral heterogeneity in three cases. In case A, 50% of cells showed high-level amplification and nonamplified cells. In addition, this case showed discordant data between IHC and qRT-PCR methodologies, probably due to qRT-PCR false-positive results. The major question is whether cells showing different levels of amplification make any difference or whether a threshold (or its value) percentage of amplified tumor cells is required to define nonamplified and amplified tumors. Regardless of the CISH results, these three patients were treated with tamoxifen and radiotherapy (patients A and B) and radiotherapy and CMF (patient C), according to the IHC results. The outcome was favorable for more than 45 months. Further studies should be performed to clarify the tumoral heterogeneity involving *HER-2 *amplifications in breast cancer. These three cases were evaluated by FISH and similar results were observed between CISH and FISH analysis for all the cases. When using FISH, it was not possible to determine the two areas observed by CISH, probably due to the restricted number of cells evaluated. Case A presented a higher frequency of amplified cells by CISH in its area 2 than that observed by FISH. Specifically in this case, the paraffin sections used for CISH and FISH methods were not successive and, most likely, different patterns of tumor heterogeneity were evaluated by both methodologies.

Comparison between CISH and IHC results revealed six discordant cases (16%). These same cases showed concordance when comparing CISH and qRT-PCR data. Many studies have shown high concordance between IHC and CISH; frequently, the discordant cases were 2+ immunostained [27,30-32]. In the present study, all of the samples scored as 3+ by IHC presented *HER-2 *amplification by CISH. In fact, >90% of HER-2 IHC 3+ tumors present *HER-2 *gene amplification [19,33]. The concordance between both methodologies was similar to that observed in other studies, varying from 85% to 95.3% [24,27,34]. Differences exist in tissue screening between these two techniques. While the IHC test requires that a minimum of 10% of tumor cells are reactive to be considered positive, CISH scoring requires that more than 50% of tumor cells show an increase in gene copies to be considered amplified; according the criteria used in the present study. It is conceivable that this difference could account for some of the discordant results observed between IHC and CISH. In the present study, eight cases presenting 2+ and 3+ immunostaining showed *HER-2 *amplification by FISH. The two cases detected as 2+ by IHC and amplified by FISH also presented high-level amplification using CISH. Cases scored as 2+ and amplified by FISH were also observed in other studies [10,35,36].

A significant correlation was detected between gene and protein expression levels. These data are in agreement with several reports that showed good correlation between transcript and protein data [37-40]. However, ten samples scored as 0 or 1+ by IHC showed *HER-2 *overexpression by qRT-PCR. The transcript quantitative analysis revealed two classes of *HER-2 *overexpression: five cases showed a median RQ = 4.09 ± 4.33 and four of these showed gene amplification by CISH; and five other cases presented a median RQ = 2.19 ± 0.21, a value very close to the cut-off value selected (2.00). Tse et al. [39] used a cut-off of RQ>2.2 as positive for *HER-2 *overexpression by qRT-PCR when it was compared to Elisa, IHC, and FISH on sections obtained from paraffin-embedded breast carcinomas.

Currently, the most widely used assay to evaluate gene status in cases scored as 2+ immunostaining is FISH. However, qRT-PCR has emerged as a potential alternative technique for assessing *HER-2 *status. The present results demonstrated that 58.3% of the samples presenting a score of 2+ overexpressed the transcript. Among the current samples, four out of five patients that presented discordant results (2+ immunostaining and RQ ≤ 2.00) showed a favorable outcome, indicating that *HER-2 *status by qRT-PCR could be performed on 2+ staining tumors with potential value regarding the management of these patients. Of the patients presenting 2+ staining and RQ>2.00 (eight cases), three presented metastasis, one revealing spreading to the bone and two to the lung.

A higher concordance rate was observed between 3+ score by IHC and overexpression by qRT-PCR. Only one sample that presented 3+ status and *HER-2 *amplification showed transcript downexpression. This case, showing a qRT-PCR false-negative result, probably resulted from the dilution of cells carrying amplified genes among nontumor cells [41], although the sample was submitted to microdissection.

Concordance between the IHC and qRT-PCR results was 78.9%. The discordance between these methodologies could include differences in the specimen used in the experiments (paraffin-embedded and fresh tumor tissue, respectively). Two other possible causes for discrepancy exist: intraobserver error, due to subjectivity of the IHC interpretation; and qRT-PCR analysis, which can cause discrepancies particularly in the initial cycles, which depend not only on the melting temperature of the amplicon, but also on the behavior of the genomic vicinity of the amplicon [42,43]. Using PCR-based methods, the expression of tumor- or tissue-specific genes and the presence of genetic abnormalities can be detected in a clinical specimen with higher sensitivity (one malignant cell out of 10^6^–10^7 ^normal cells) than that of other techniques such as light microscopy (one malignant cell out of 10^2^–10^3 ^normal cells). Using RT-PCR the nucleic acid molecules can be amplified 10^10^-fold [for review, [44]]. Moreover, *HER-2 *overexpression can be detected in 0.1 cell equivalent spiked into 8 mL of peripheral blood using qRT-PCR and the detection limited increases to 10 and 50 cell equivalent per 8 mL in cell lines expressing intermediate and low levels of *HER-2 *[45].

Comparison between qRT-PCR data and CISH results showed that the gene expression median was correlated with gene copy number, a finding also observed by Bergqvist et al. [46]. *HER-2 *gene amplification is the most prevalent genetic mechanism driving *HER-2 *overexpression. The discordant cases (8.1%) showed 2+ and 3+ immunostaining, confirming the CISH results. In this study, the concordance between qRT-PCR and FISH was 100%. High concordances were observed by other studies [23,47].

In the present study, a high correlation rate among the three procedures used to score *HER-2 *status in breast carcinomas was observed. The correlation between CISH and qRT-PCR was higher than CISH and IHC, which was higher than qRT-PCR and IHC for the samples evaluated by all these procedures. In fact, CISH and qRT-PCR are complementary methodologies for evaluating *HER-2 *status.

No correlation was found between HER-2 expression by IHC or gene copy number and clinicopathological data. However, a significant association was observed between lymph node status and *HER-2 *transcript expression by qRT-PCR. Peiró et al. [48] analyzed HER-2 status by IHC and CISH and observed a correlation with histological grade and lymph-vascular invasion, but no association was found with age, tumor size and Ki-67 status. Similarly, the absence of correlation between HER-2 status and clinical and pathological features has been reported in other studies [32,43].

No statistically significant difference was observed between ductal and lobular carcinomas evaluated by qRT-PCR. Among the lobular carcinomas, amplification by CISH was observed in one case, followed by *HER-2 *overexpression and negative IHC staining. In a previous study, this case was confirmed as negative for e-cadherin protein expression and presented *CDH1 *promoter hypermethylation [49]. Li-Ning-T et al. [35] evaluated five invasive lobular tumors and none showed amplification by CISH, however two cases presented 2+ immunostaining.

## Conclusion

In conclusion, the present results suggest that *HER-2 *status can be performed by CISH and qRT-PCR analysis. CISH combines the advantages of IHC and FISH and is a promising practical alternative to FISH, while qRT-PCR is reliable, semiautomated and fast; and both methodologies can be performed in most pathology laboratories. However, there are several limitations to apply qRT-PCR as a routine method for clinical application, including the use of fresh frozen tissue, microdissection procedures and the incapacity to address cell-to-cell variations. In addition, the data confirm that *HER-2 *overexpression is associated with a worst prognostic in human breast tumors.

## Competing interests

The authors declare that they have no competing interests.

## Authors' contributions

FER participated in the study design, in defining the casuistic used, carried out the qRT-PCR analysis and helped to draft the manuscript. SMS, CGTS, and NAB performed the CISH analysis. FAMN, MACD, and FAS carried out the pathological and immunohistochemistry analysis. JRFC provided the samples and follow-up data. SRR conceived the study, participated in its design, performed the statistical analysis and helped to draft the manuscript. All authors read and approved the final manuscript.

## Pre-publication history

The pre-publication history for this paper can be accessed here:

http://www.biomedcentral.com/1471-2407/9/90/prepub
